# 5-Lipoxygenase contributes to PPARγ activation in macrophages in response to apoptotic cells

**DOI:** 10.1186/cc11770

**Published:** 2012-11-14

**Authors:** A von Knethen, L Eifler, L Kuchler, A Heeg, H Heide, I Wittig, T Maier, D Steinhilber, B Brüne

**Affiliations:** 1 Institute of Biochemistry I Faculty of Medicine, Goethe-University Frankfurt, Germany; 2 Faculty of Medicine, Goethe-University Frankfurt, Germany; 3 Institute of Pharmaceutical Chemistry, Goethe-University Frankfurt, Germany

## Background

One hallmark contributing to immune suppression during the late phase of sepsis is macrophage polarization to an anti-inflammatory phenotype upon contact with apoptotic cells (AC). Taking the important role of the nuclear receptor PPARγ for this phenotype switch into consideration, it remains elusive how AC activate PPARγ in macrophages. Therefore, we were interested to characterize the underlying principle.

## Methods

Apoptosis was induced by treatment of Jurkat T cells for 3 hours with 0.5 μg/ml staurosporine. Necrotic cells (NC) were prepared by heating cells for 20 minutes to 65°C. PPARγ activation was followed by stably transducing RAW264.7 macrophages with a vector encoding the red fluorescent protein mRuby after PPARγ binding to 4 × PPRE sites downstream of the reporter gene sequence. This readout was established by treatment with the PPARγ agonist rosiglitazone (1 μM) and AC (5:1). Twenty-four hours after stimulation, mRuby expression was analysed by fluorescence microscopy. Lipid rafts of AC, NC, as well as living cells (LC) were enriched by sucrose gradient centrifugation. Fractions were analysed for lipid raft-associated marker proteins. Lipid rafts were incubated with transduced RAW264.7 macrophages as described above. 5-Lipoxygenase (5-LO) involvement was verified by pharmacological inhibition (MK-866, 1 μM) and overexpression.

## Results

Assuming that the molecule responsible for PPARγ activation in macrophages is localized in the cell membrane of AC, most probably associated to lipid rafts, we isolated lipid rafts from AC, NC and LC. Mass spectrometric analysis of lipid rafts of AC showed the expression of 5-LO, whereas lipid rafts of LC did not. Moreover, incubating macrophages with lipid rafts of AC induced mRuby expression. In contrast, lipid rafts of NC and LC did not. To verify the involvement of 5-LO in activating PPARγ in macrophages, Jurkat T cells were incubated for 30 minutes with the 5-LO inhibitor MK-866 (1 μM) before apoptosis induction. In line with our hypothesis, these AC did not induce mRuby expression. Finally, although living Jurkat T cells overexpressing 5-LO did not activate PPARγ in macrophages, mRuby expression was significantly increased when AC were generated from 5-LO overexpressing compared with wild-type Jurkat cells.

## Conclusion

Our results suggest that induction of apoptosis activates 5-LO, localizing to lipid rafts, necessary for PPARγ activation in macrophages. Therefore, it will be challenging to determine whether 5-LO activity in AC, generated from other cell types, correlates with PPARγ activation, contributing to an immune-suppressed phenotype in macrophages.

